# Searching for a Solar Source of Magnetic-Field Switchbacks in Parker Solar Probe’s First Encounter

**DOI:** 10.1007/s11207-022-02022-4

**Published:** 2022-07-15

**Authors:** D. de Pablos, T. Samanta, S. T. Badman, C. Schwanitz, S. M. Bahauddin, L. K. Harra, G. Petrie, C. Mac Cormack, C. H. Mandrini, N. E. Raouafi, V. Martinez Pillet, M. Velli

**Affiliations:** 1 Mullard Space Science Laboratory, University College London, Holmbury St. Mary, Surrey, RH5 6NT UK; 2 Indian Institute of Astrophysics, Koramangala Bangalore, 560034 India; 3 Physics Department, University of California, Berkeley, CA 94720-7300 USA; 4 Space Sciences Laboratory, University of California, Berkeley, CA 94720-7450 USA; 5 PMOD/WRC, Davos-Dorf Davos, CH-7260 Switzerland; 6 Instituto de Astronomía y Física del Espacio, IAFE, UBA-CONICET, Buenos Aires, Argentina; 7 ETH-Zürich, Hönggerberg campus, HIT building, Zürich, Switzerland; 8 Laboratory for Atmospheric and Space Physics, University of Colorado, Boulder, CO 80303 USA; 9 National Solar Observatory, 3665 Discovery Drive, Boulder, CO 80303 USA; 10 The Johns Hopkins Applied Physics Laboratory, Laurel, MD 20723-6099 USA; 11 Department of Earth, Planetary, and Space Sciences, University of California, Los Angeles, Los Angeles, CA USA

**Keywords:** Solar wind, Coronal holes, Observations

## Abstract

Parker Solar Probe observations show ubiquitous magnetic-field reversals closer to the Sun, often referred to as “switchbacks”. The switchbacks have been observed before in the solar wind near 1 AU and beyond, but their occurrence was historically rare. PSP measurements below ∼ 0.2 AU show that switchbacks are, however, the most prominent structures in the “young” solar wind. In this work, we analyze remote-sensing observations of a small equatorial coronal hole to which PSP was connected during the perihelion of Encounter 1. We investigate whether some of the switchbacks captured during the encounter were of coronal origin by correlating common switchback in situ signatures with remote observations of their expected coronal footpoint. We find strong evidence that timescales present in the corona are relevant to the outflowing, switchback-filled solar wind, as illustrated by strong linear correlation. We also determine that spatial analysis of the observed region is optimal, as the implied average solar-wind speed more closely matches that observed by PSP at the time. We observe that hemispherical structures are strongly correlated with the radial proton velocity and the mass flux in the solar wind. The above findings suggest that a subpopulation of the switchbacks are seeded at the corona and travel into interplanetary space.

## Introduction

The presence of ubiquitous magnetic “switchbacks” below ∼ 0.2 AU was one of the most surprising discoveries by *Parker Solar Probe* (PSP: Fox et al., 2016). These features included sudden rotations of the magnetic-field vector in solar-wind observations by up to 180^∘^ (Bale et al., 2019), and were often accompanied by velocity jets (Kasper et al., 2019). The correlation between these magnetic and velocity disturbances are typically highly Alfvénic (Tenerani et al., 2020). Sunward electron strahl measurements show that the field reversals are not consistent with polarity changes (i.e., current sheets), but instead smooth deformations of the field in a switchback shape (Kasper et al., 2019).

Since their discovery, various models of possible generation mechanisms for these switchbacks have been proposed. Broadly, these mechanisms may be divided into two categories: 1) Those of coronal origin where the switchbacks are generated by transient processes in the solar atmosphere and subsequently injected onto open field lines where they travel out into the heliosphere; 2) Those of in situ generation within the solar wind, whereby the ambient plasma conditions lead to instabilities or wave steepening that give rise to the natural formation of switchbacks without a specific impulsive root.

Examples of formation models in the coronal origin category mostly recall interchange magnetic reconnection between closed and open field lines (Fisk and Schwadron, 2001; Fisk and Kasper, 2020). Different authors have investigated how this fundamental process in the corona can lead to propagating “S-shape” disturbances in the Alfvén mode (Tenerani et al., 2020), fast magnetosonic mode (Zank et al., 2020), or as a flux rope (Drake et al., 2021). These different models have distinct strengths in terms of fitting the in situ observations and in generating switchbacks sufficiently stable to propagate significant distances in the solar wind, but all produce the observable expectation that a transient event at the Sun has the ability to lead to a switchback-like disturbance reaching PSP. Other studies have suggested coronal jets as potential sources of Alfvénic disturbances, which could constitute switchbacks and escape the corona (e.g., Sterling and Moore, 2020; Sterling et al., 2020).

On the other hand, other models have been proposed in which switchbacks arise “naturally” in the solar wind, given certain ambient conditions. Squire, Chandran, and Meyrand (2020) found switchback-like disturbances forming within magnetohydrodynamic (MHD) simulations as a natural consequence of the growth of small-amplitude Alfvénic perturbations in the expanding solar wind. A recently submitted follow on paper by Mallet et al. (2021) extended this analysis to address other compelling observable features such as the switchbacks being predominantly field-aligned elongated structures (Horbury et al., 2020; Laker et al., 2021) and that they occur in patches (Bale et al., 2019). Another in situ origin theory (Schwadron and McComas, 2021) posits a simple geometric explanation in which a slow solar-wind stream is leading a fast solar-wind stream, forcing the shape of the slow stream’s spiral field line into a switchback beyond the Alfvén critical surface.

In a recent development, Fargette et al. (2021) and Bale et al. (2021) have found a correlation between the switchback patches and spatial movement of the magnetic footpoints in the photosphere. Combined with analysis of differing plasma properties within the patches, these authors suggest patches of switchbacks are tied to supergranular angular scales at the solar surface. While this result does not resolve the in situ or coronal origin conundrum, it suggests switchbacks are generated within specific streams, which are in turn formed by magnetic reorganization in the lower corona. This demonstrates that solar-wind sources in the corona are playing a role in switchback formation. Since these streams do form between open and closed field boundaries, they further motivate the idea that switchbacks could be formed in the corona by interchange reconnection.

In this work, we aim to find direct evidence of the coronal influence on switchbacks through the study of data captured during PSP’s first solar encounter, as the spacecraft was connected to an equatorial coronal hole (CH) (see, e.g., Bale et al., 2019; Badman et al., 2020).

## Observations and Data Analysis

**Figure 1:**
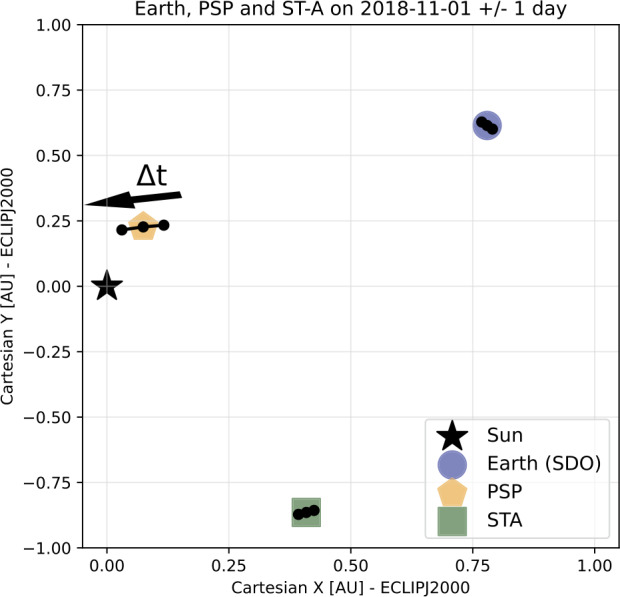
Orbital positions of the Sun, Parker Solar Probe, and the STEREO-Ahead spacecraft on 1 November in the ECLIPJ2000 frame. The *black dots* indicate the body position the day before and after. The *black arrow* above PSP indicates the direction of motion of the spacecraft.

To compare coronal measurements from the region where the solar wind originates to in situ measurements of the stream sampled during the first PSP encounter, we collected available remote and in situ data that were relevant. We show in Figure 1 the relative positions of Earth, STEREO-Ahead, and the Parker Solar Probe spacecraft.

### In Situ Observations

All in situ observations used in this article are from the PSP spacecraft. We collected variables that are observed to vary within switchbacks, as well as variables that help us understand the nature of the solar-wind stream. We used the 1-minute cadence magnetic-field data measured by the Electromagnetic Fields Investigation flux-gate magnetometers (FIELDS: Bale et al., 2016), as well as the plasma parameters, including radial proton velocity, proton density, and temperature, available from 08:00 UT on 31 October from the Solar Wind Electrons, Alphas and Protons (SWEAP: Kasper et al., 2016). The solar-wind mass flux, M_f_ was calculated, M$_{\mathrm{f}} = n_{\mathrm{p}}$ $\times \sqrt{V_{\mathrm{x}}^{2} + V_{\mathrm{y}}^{2} + V_{\mathrm{z}}^{2}}$, which is representative of mass loss from the solar atmosphere and depends on the proton number density, and the magnitude of the bulk-flow velocity.

**Figure 2:**
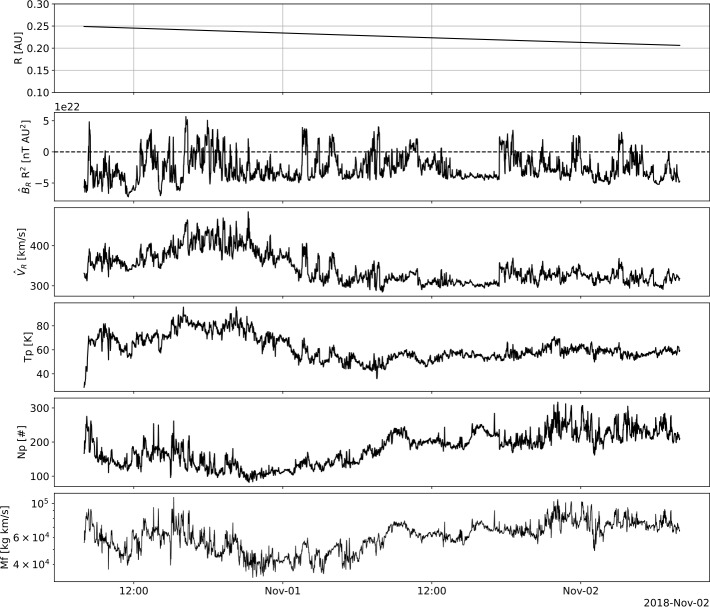
PSP measurements used for correlation in this work. From top to bottom we show the radial distance to the solar surface, scaled radial magnetic field, radial proton velocity, proton temperature, proton-number density, and mass flux.

Figure 2 shows the 48 hours of 1-minute PSP measurements that were used for our correlation analysis. The spacecraft distance to the solar surface, the scaled radial magnetic field, the radial velocity, the proton temperature, the proton density, and the mass flux are shown from top to bottom, respectively. We identified the switchbacks as measurement intervals when the scaled radial magnetic-field component became positive during the predominantly negative polarity field.

We derived the PSP orbital positions using the available SPICE kernels and employed ballistic backmapping and *Potential Field Source Surface* (PFSS; Altschuler and Newkirk, 1969; Schatten, Wilcox, and Ness, 1969; Wang and Sheeley, 1992) modeling to determine the likely solar footpoints of the stream. Using the average proton velocity measured in PSP of 342 km s^−1^ as an indicator for the propagation speed of features from the corona to the spacecraft, we calculated the expected emission time and collected remote observations, which contextualised and described the coronal activity in the region.

**Figure 3:**
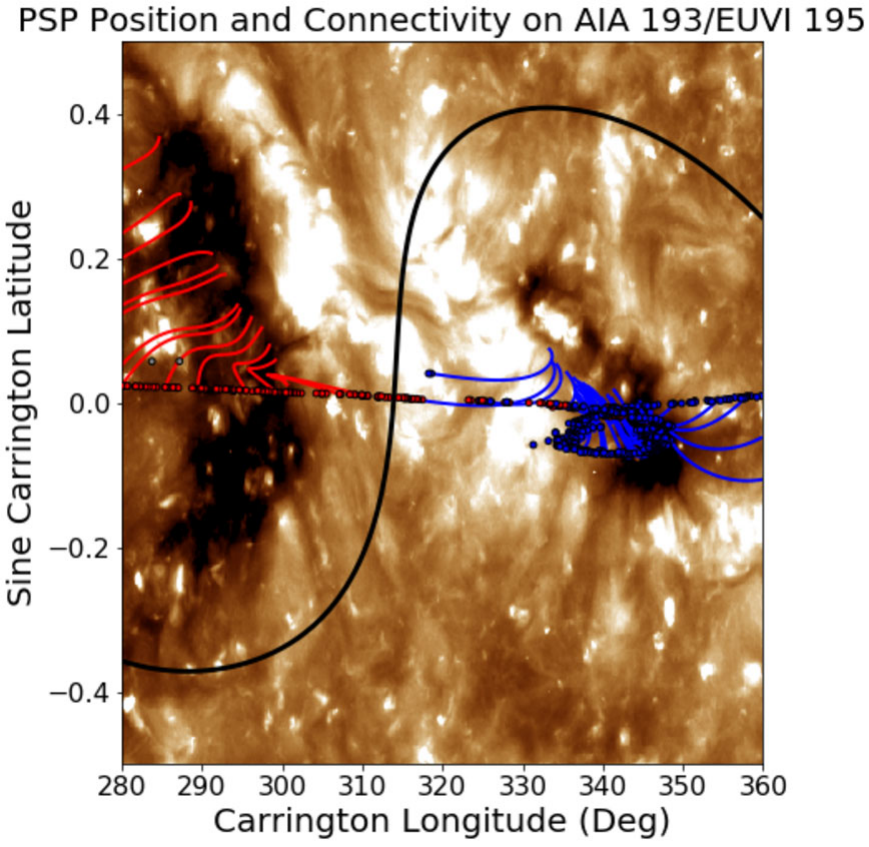
*Blue and red* scatter points show PSP’s trajectory ballistically backmapped to 2.0 R_⊙_ for its first solar Encounter, with *blue* (*red*) indicating negative (positive) polarity in situ. Field lines connect the backmapped trajectory to solar sources via a PFSS model, colored by their polarity in the model. A *solid black line* indicates the PFSS model heliospheric current sheet. The background image shows a combined Extreme Ultraviolet Imager (EUVI: Wuelser et al., 2004) 195-Å and Atmospheric Imaging Assembly (AIA: Lemen et al., 2012) 193-Å synoptic map for Carrington Rotation (CR) 2210. The AIA instrument is onboard the Solar Dynamics Observatory (SDO: Lemen et al., 2012) PSP is seen to connect primarily to a small negative-polarity equatorial coronal hole (centred at CR longitude 345 ^∘^ in the background image) throughout its corotation loop (see also Bale et al., 2019; Badman et al., 2020).

We show in Figure 3 the PFSS-calculated footpoints for the PSP positions during the encounter, with the dots indicating the prevailing polarity detected in PSP, the magnetic-field lines being colored depending upon the predicted photospheric polarity, and the black line indicating the heliospheric current sheet. The small CH is positioned between 330 and 350^∘^ within Figure 3, and shows predominantly negative polarity, which is in broad agreement with the colored dots, and hence implies that it is likely PSP was sampling solar wind from this region.

### Remote-Sensing Data and Coronal Modeling

We selected remote-sensing observations for context or direct comparison based on availability. We begin by explaining the observations that provide context and then present those used for crosscorrelation in the temporal or the spatial domain. The “small” CH was present on the solar disc during six Carrington rotations (CR2206 – CR2211), from 09 July to 10 December 2018, with varying extension and morphology. CR2210 extends from 26 October to 23 November 2018 during the PSP in situ data time period used in our correlation analysis. However, we show the global coronal models for both CR2209 (29 September to 26 October 2018) and CR2210 as PSP Encounter 1 overlaps half of CR2209 and all of CR2210 (Badman et al., 2020).

To visualize the large-scale coronal connectivity in its surroundings, we used global PFSS extrapolations for all CRs for which the small CH was seen. We used Helioseismic Magnetic Imager (HMI: Scherrer et al., 2012) synoptic maps as the boundary condition. Our models are based on the Finite Difference Iterative Potential-Field Solver (FDIPS) code (Tóth, van der Holst, and Huang, 2011). This code, freely available from the Center for Space Environment Modeling (CSEM) at the University of Michigan (csem.engin.umich.edu/tools/FDIPS), solves the Laplace equation for the magnetic field using an iterative finite-difference method. The spatial resolution for our particular models is $1^{\circ}$ in longitude (360 longitudinal grid points), $1.1\times 10^{-2}$ in the sine of latitude (180 latitudinal grid points) and 1×10^−2^ $\mathrm{R}_{\odot}$ in the radial direction. Our models assume that the field becomes purely radial at a height, called the source surface, which is set to the value $2.5\,\mathrm{R}_{\odot}$.

**Figure 4:**
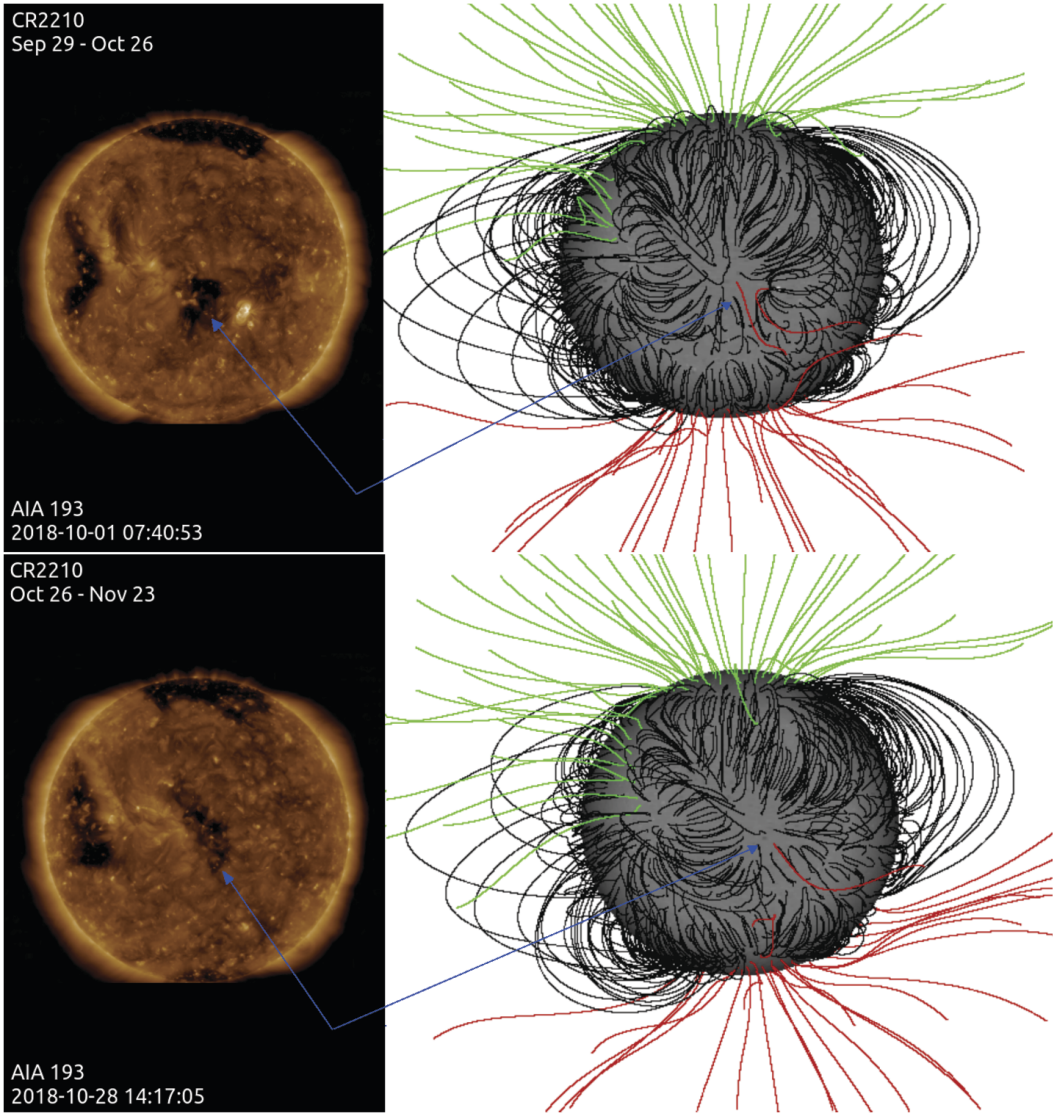
*Left*: AIA 193-Å full-disc images when the small CH was located at the central meridian passage on CR 2209 (*top*) and CR 2210 (*bottom*). Right: PFSS models with the CH at a similar location as in the AIA images (Carrington longitude 335^∘^) for CR 2209 (*top*) and CR 2210 (*bottom*). In *black* we have drawn closed field lines, while in *red (green)* we show open field lines anchored in negative (positive) field polarities. The *blue arrows* point to the approximate location of the small CH both in AIA images and models.

In the top-left panel of Figure 4 we show the CH at central meridian passage in AIA 193 Å on CR2009, while at the bottom left a similar image corresponds to CR2210. The right panels depict the reconstruction of the potential magnetic field with the CH facing Earth. The variation in size and shape of the small CH is clear when comparing the two AIA full-disc images.

**Figure 5:**
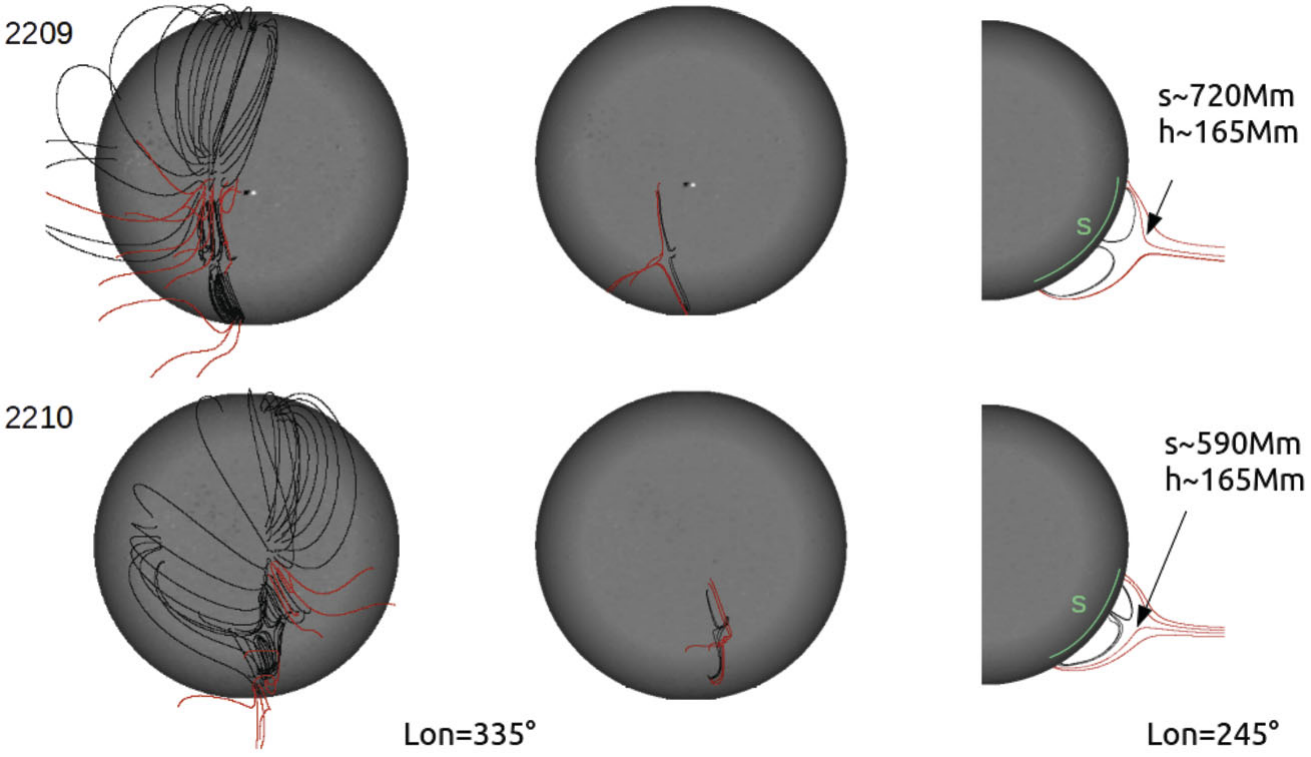
*Top* model corresponds to CR2209 and *bottom* one to CR2210. To the left we show a selected set of field lines anchored to the small CH, its surrounding, and the southern CH; in these models the solar-disc center is located at a longitude of $335^{\circ}$. The *central* and *right* models depict the same reduced set of field lines illustrating the structure of the pseudostreamer as seen from the same point of view as in the *left* models and when the small CH is at the western solar limb (see text for the distances indicated in the *right* models). The field-line color convention is the same as in Figure 4.

During CR2209 and CR2210, a set of field lines anchored to the south of the small CH formed a pseudostreamer together with field lines from the southern CH; PSP’s first solar encounter spanned the transition between these two Carrington rotations. Figure 5, to the left and center, shows a selected set of field lines anchored in the small CH, its surroundings, and also in the southern CH. The top PFSS model corresponds to CR2209 and the bottom one to CR2210. In the left and central figures, the solar-disc center is located at a longitude of $335^{\circ}$. The central and right models depict the same and more reduced number of field lines to illustrate the shape of the pseudostreamer when the small CH was located as in the left models and in the western solar limb. We estimate a height of ≈ 165 Mm for the transition between closed and open magnetic-field lines. The distance over the disc between the outer edges of the pseudostreamer is $s \approx $ 720 Mm and 590 Mm for CR2209 and CR2210, respectively. It is clear from these models that the pseudostreamer associated with the small CH was a quite stable structure. This is important since PSP mostly connected to this structure while behind the limb when synchronous remote observations were unavailable; however, it is clear from this modeling that the structure existed both before and after (and therefore “during”) PSP’s time of connectivity.

**Figure 6:**
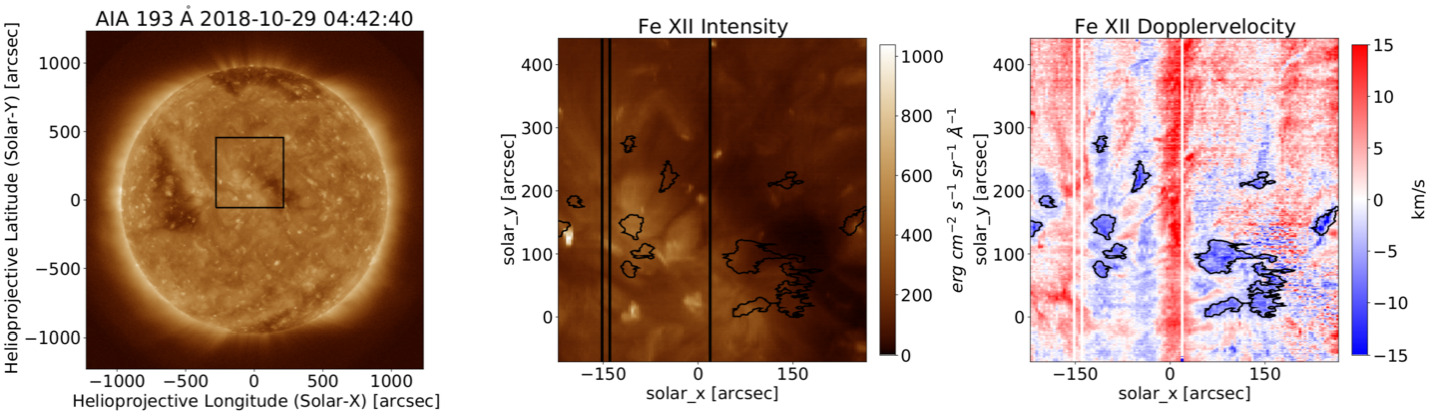
A Hinode/EIS raster of the small CH on the 29 October 2018 at 04:42:42 UT, 195.12 Å. The *left panel* shows the EIS field-of-view on the disc. The *central panel* shows the intensity of the Fe xii line. The *right panel* shows the Doppler velocities derived from the Fe xii line. 13 regions of blueshifts stronger than -5 km s^−1^ are highlighted. They are equally distributed at the small CH boundary and on the quiet Sun.

The EUV Imaging Spectrometer (EIS) on board Hinode carried out raster scans of the CH on the 29 October 2018 at 04:42:42 UT. Plasma released on open field lines from the structure at this time would be expected to reach PSP’s radial distances at a time when it connected to this CH although, as explored in this work, there are a variety of assumptions that can be made about the transit time that prove important for our correlative studies. The raster covers the northern region of the small CH with a FOV of $492''\times 512''$. This raster is analyzed in detail to provide a general understanding of the coronal upflow regions within. The Fe xii emission line at 192.15 Å is used to derive Doppler velocities. The Doppler velocities are then used to identify regions with upflows stronger than −5 km s^−1^ and these are displayed in Figure 6. Only regions with an area of more than 15 pixels are considered, which results in 13 upflow regions. Six of those regions are located at the small CH boundary and seven are in the quiet Sun. Furthermore, seven regions are in, or intersect with, bright points. They have different shapes, from highly elongated to circular, and complex shapes. The smallest spatial area of coronal upflow is 261 arcsec^2^ and the largest one 2747 arcsec^2^.

We analyzed AIA observations to understand the sources of the upflows (i.e., blueshifts). The data of the 193-Å channel within the blueshift regions shows intensity enhancements (decreases) for seven (six) areas. A connection to their location or bright points could not be made. However, movies made from AIA data reveal clear causes for two out of the 13 regions. One is caused by the eruption of a small loop, which shows an outflow. The second region shows a clear brightening. A potential outflow might be concealed by foreground coronal haze and is not observed. The analysis of the spectral raster and the derived blueshifts show that most upflow regions are not caused by transient activity, but probably by longer existing structures. Unfortunately, no consecutive rasters were taken, which prevents us from understanding the more permanent upflow regions better.

To quantify the open magnetic flux associated with the small CH over time, we used HMI magnetograms together with AIA 193-Å images, both at a 12-minute cadence. The small CH boundaries were identified by constructing a histogram of AIA 193-Å pixel-by-pixel intensity values, which is dominated by a main peak representing the general quiet-Sun intensity values and a dark tail representing the small CH intensity values. The small CH boundary intensity could be defined as the value where the dark histogram tail meets the main peak. For each AIA image, we computed contour curves with this intensity value I_CHB_, and used the curves to mask the corresponding HMI magnetogram.

Photospheric magnetic flux is dominated by small flux concentrations at supergranular boundaries, of size ≈ 5 – 10 arcsec, as observed from near Earth. These flux concentrations emerge as bipoles (i.e., pairs of opposite-polarity concentrations) and disappear when they meet and cancel with concentrations of opposite polarity. The photospheric-flux distribution may therefore change the topology of the coronal field, opening it up or closing it down, via the emergence or cancellation of bipoles and the introduction or removal of associated closed-loop structure. This supergranular structure has recently been linked to switchback patches observed by PSP (Fargette et al., 2021; Bale et al., 2021) and so are critical to study. To determine the temporal evolution of bipole flux in and near the CH over time, we used the YAFTA (Yet Another Feature-Tracking Algorithm) feature-tracking code (Welsch and Longcope, 2003). The bipole flux is plotted against time in the top panel of Figure 8. A 1-hour boxcar filter was applied to the bipole flux (solid curve) to make the visual comparison clearer.

**Figure 7:**
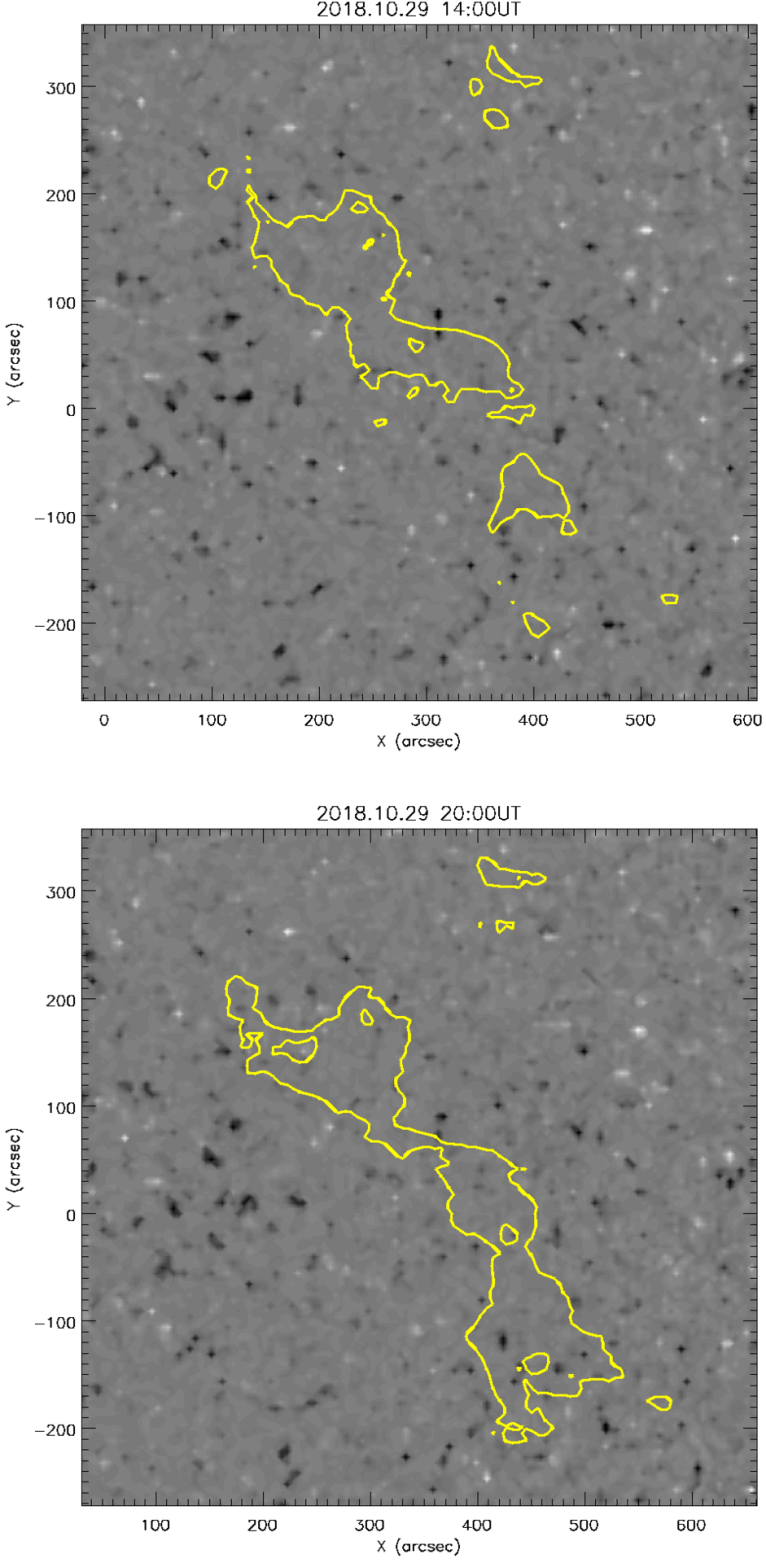
Two snapshots of the small CH evolution, six hours apart, before and after the abrupt growth phase. The AIA small CH boundary contours are overplotted on the corresponding HMI photospheric magnetogram. Positive/negative flux density is represented by *light/dark grayscale*, saturated at 100 G.

Figure 7 shows two snapshots of the small CH evolution, six hours apart. In each panel, the AIA small CH boundary contours are overplotted on the corresponding HMI magnetogram. The plots are presented in image-plane coordinates, but the HMI magnetic field has been divided through by the cosine of the heliospheric angle to give an estimate of the radial flux density, assuming the quiet-Sun photospheric field to be approximately radial. The figure shows the degree to which the small CH grew within a few hours.

**Figure 8:**
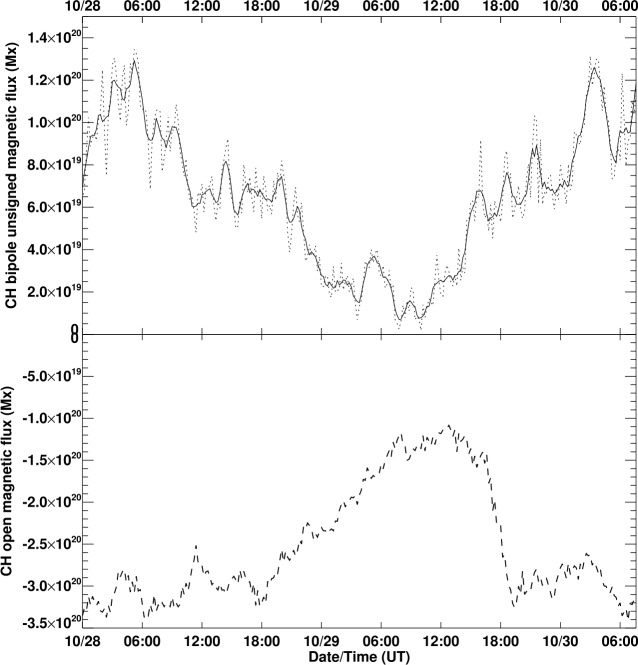
Total unsigned magnetic flux (*top*) and open magnetic flux (*bottom*) associated with the small CH, plotted against time. In the *top panel* the 12-minute data are plotted as a *dotted line* and 1 hour boxcar-filtered data are plotted as a *solid line*.

The resulting CH flux integrated over this closed contour is plotted against time in the bottom panel of Figure 8. This curve shows a simple pattern of behavior, holding a steady value of $\approx -3\times 10^{20}$ Mx for the first 18 hours of 28 October, then decreasing its absolute value steadily to $\approx -1.2\times 10^{20}$ Mx over the next 12 – 18 hours, before growing abruptly back to $\approx -3\times 10^{20}$ Mx between about 16:00 UT and 20:00 UT on 29 October (notice that the top and bottom panels of Figure 7 correspond to the time before and after the abrupt change in open flux) and remaining around this value until the data become less reliable on 30 October as the small CH region approaches the solar west limb. It is important to note that while the small CH persisted through to the next CR (see Figure 5), these results show it was highly dynamic and likely continues to be dynamic after limb passage and during much of the time that PSP is connected to it.

Shortly before the abrupt CH open-flux absolute value increased by a factor > 2 around 18:00 UT on 29 October, as shown in the bottom panel of Figure 8, the top panel of this figure shows that a total bipole flux change occurred that was, if anything, more spectacular, albeit not so simple. Between about 10:00 UT and 16:00 UT, the total unsigned flux increased by an order of magnitude. This change may be relevant throughout the link between photospheric bipoles and coronal bright points and jets that may in turn be relevant to the coronal magnetic switchbacks. Such a large and abrupt change in the bipoles associated with a CH might be worth investigating in more granular detail.

Of course, the correlation between the CH open flux and the total unsigned flux is mostly due to the common CH mask derived to estimate the two quantities, but there are also differences. The significant time lag of ≈ 3.5 hours of the open-flux change relative to the total bipole flux change suggests that the magnetic complexity of the CH increased a few hours ahead of the open-flux abrupt change. The open flux has a higher floor than the total bipole flux, and the open-flux change was simpler and more abrupt. The open-flux evolution closely follows the CH area changes, whereas the bipole flux follows the number of bipoles. The bipole population changes led the CH area changes by a few hours, perhaps suggesting a causal link between the two.

**Figure 9:**
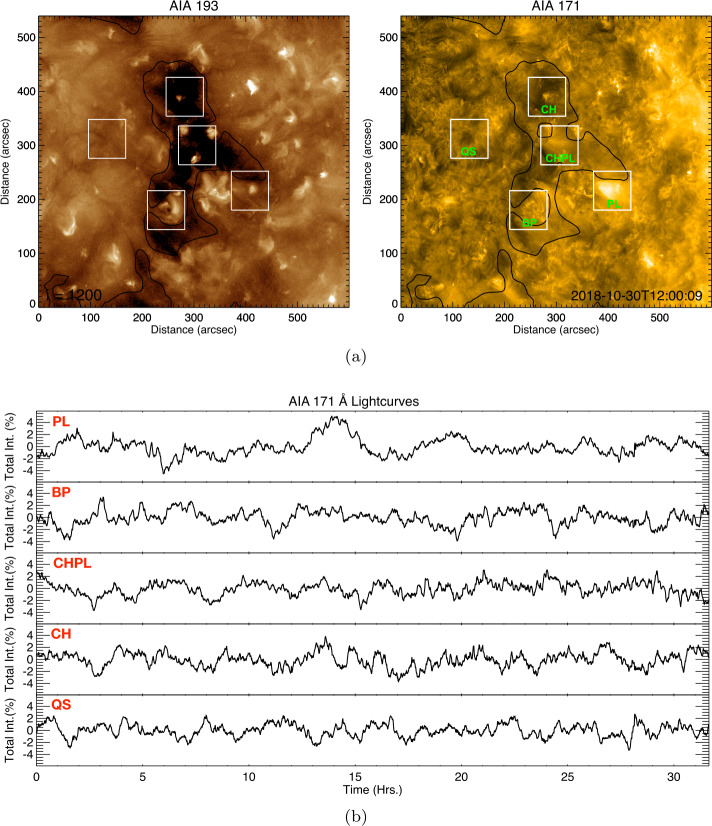
a) Small CH in SDO/AIA 193- and 171-Å passbands, with five labeled regions: CH for coronal hole, QS for quiet Sun, BP for bright point, PL for plume, and CHPL for coronal-hole plume. b) Detrended lightcurves extracted from each of the regions shown in *panel* (*a*) for the 171-Å passband, showing hours since 12:00 UT 30 October 2020. From top to bottom we show the plume, bright point, coronal-hole plume, coronal hole, and quiet-Sun regions.

In addition to the photospheric observations, we extracted five distinct regions around the expected PSP solar footpoints, being either rooted in the small CH, or in one of the structures around it. In Figure 9(a) we show these five selected regions in the upper corona as imaged by the SDO/AIA 171-Å passband. The purpose of the 171-Å passband was twofold, for it was additionally used to characterize the quiet corona and upper transition region in the analysis. We used SDO/AIA 193-Å observations to characterize hotter plasma observed in the corona and provide a more comprehensive basis for the comparison to the solar wind. The five regions were selected to provide insights into several areas found near the calculated magnetic footpoints of the spacecraft, each of which displayed different activity levels, with the condition that dynamics present within the box at the beginning remained within the box during the 30-hour time window of the analysis. The coronal-hole region is intended to capture a low-activity portion of the small CH. The quiet-Sun region is intended to represent quiet-Sun variation. The coronal-hole plume and plume regions capture a coronal plume inside and outside the investigated small CH. The bright-point region captures an emergent bipole within the small CH. We show the resulting box-car averaged datasets in Figure 9b, using a sampling length of 200 measurements, to remove the influence of background dynamics from the extracted lightcurves.

## Analysis

As explained in Section 2, observations sourced from SDO/HMI and SDO/AIA were analyzed and directly correlated to the in situ PSP measurements for the stream rooted at the small CH. The goal of this comparison was to identify similarities in timescales and spatial frequencies present in the remote-sensing and in situ observations. We also obtained specific times and outflow coordinates at the source-surface height by averaging PSP velocity measurements to an hourly cadence, to then use ballistic backmapping to a source-surface radius of 2.0 R_⊙_ for an expected emission time.

We compared features observed in the corona and the photosphere to those observed by PSP through the use of the Pearson correlation coefficient (Freedman, Pisani, and Purves, 2007). This is a measure of linear correlation between two sets of data, defined as the covariance of the two variables divided by the product of their standard deviations. The correlation coefficient then takes a value between −1 and 1, depending on how well one dataset can be predicted by using the other. As with covariance itself, we note that it can only reflect a linear relation between the parameters, in this case in situ variables and the remotely captured lightcurves. In this paper we use a coherently structured high correlation coefficient as evidence for temporal or spatial signatures from the Sun appearing within observations at PSP.

In this section, we first attempt the temporal correlation of lightcurves from unique regions surrounding the PSP footpoints by studying 9-hour windows of observations. We then explored whether spatial frequencies in the corona were more strongly correlated to the switchback patches by applying a spatial Fourier transformation and comparing its output to the PSP measurements.

### Temporal Analysis

We first calculated the crosscorrelation of temporal windows of the SDO/AIA regional lightcurves and PSP measurements. We then utilized a signal-decomposition technique to denoise and clean the remote and in situ observations before correlating the datasets, and we finally compared SDO/HMI photospheric measurements with the PSP observations.

#### SDO/AIA – PSP Direct Crosscorrelation

**Figure 10:**
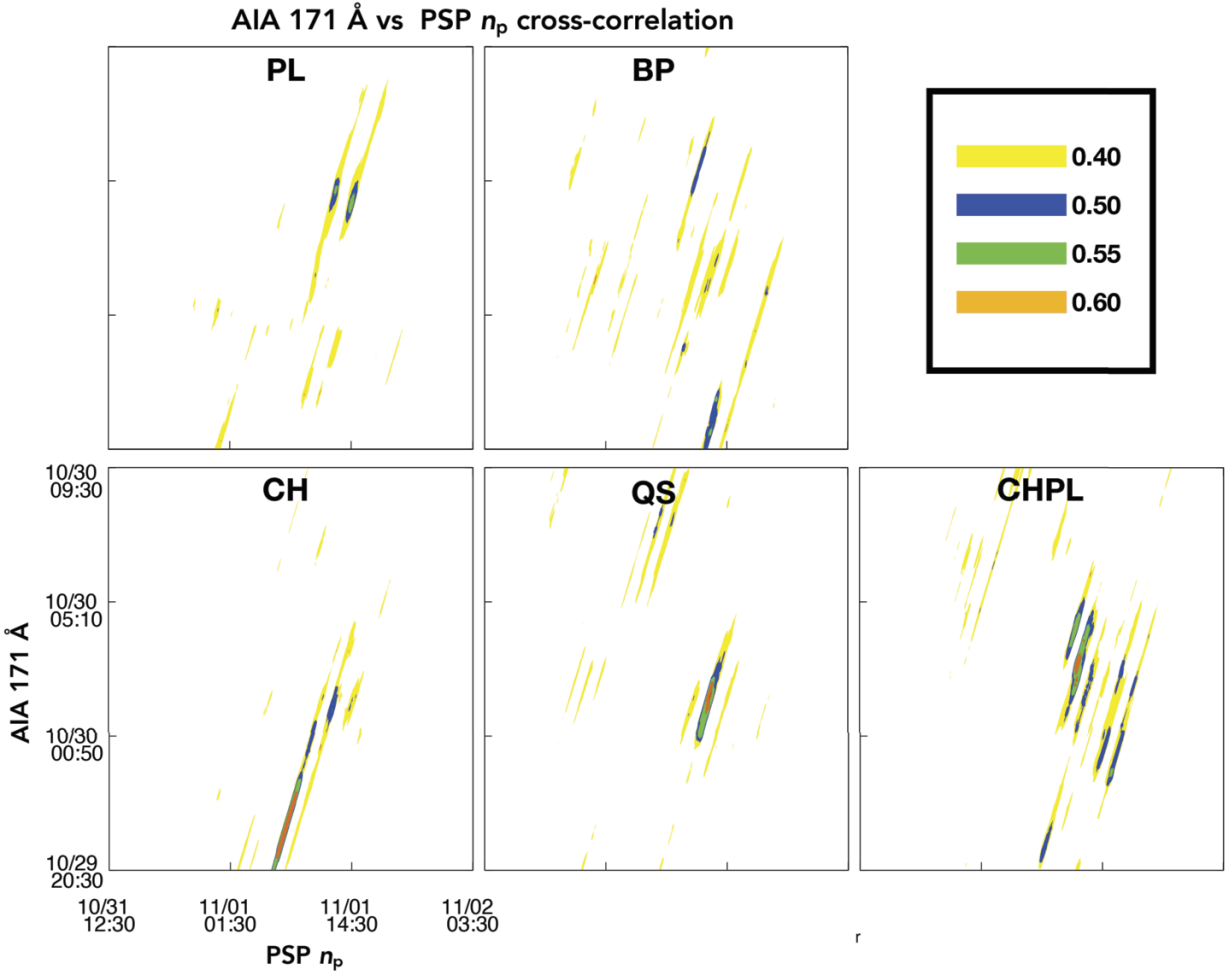
The panels show the absolute correlation coefficient between the PSP proton number density (Np, Figure 2) and the AIA 171-Å lightcurves that we show in Figure 9b. The correlation coefficients are obtained by using time windows of 9 hours from both the PSP Np and AIA 171-Å lightcurves. The maps are computed by shifting the 9-hour time windows by 1 minute in PSP Np and subsequently in the AIA 171-Å lightcurve. The time on both axes represents the midtime of the 9-hour window. The correlation coefficients of different amplitudes are marked by different colors (see the *top-right panel*). The *oblique ridges* in the plots appear due to the shifting of the 9-hour time windows in both directions (AIA and PSP) with a 1-minute cadence that eventually leads to almost a similar lightcurve with just a 1-minute shift in the diagonal space of the plot. Hence, the crosscorrelation values also become similar in the neighborhood point that creates diagonal ridges in the plot.

We extracted coronal dynamics from intensity variations in the SDO/AIA 171- and 193-Å passbands (see Figure 9). We also employed a box-car average with a window of 200 minutes to subtract the trend, and crosscorrelated the remote and in situ parameters in windows of varying temporal durations from 3 to 9 hours. We slid the relevant window to select data from the PSP radial magnetic field and each of the five coronal regions, accounting for a large range of possible ballistic propagation times from the corona to PSP. We preferentially studied the 9-hour window, which we show in Figure 10, and found that the SDO/AIA lightcurves did not indicate a strong direct correlation with PSP observations, often taking absolute values below 0.6. Stronger correlations above 0.7 were found for some of the shorter time windows (3 or 6 hours); however, upon visual inspection, there was no evident trend match of the two datasets, as the time delay between correlations appeared to be random, with no consistency across the parameter space of in situ observations. Due to the above, we analyzed the signals in more detail using more advanced signal decomposition and analysis techniques.

#### SDO/AIA – PSP Empirical Mode Decomposition Crosscorrelation

As no strong correlations were found using pure crosscorrelation, we tried a more sophisticated method that used Empirical Mode Decomposition (EMD: Huang et al., 1998) to extract temporal-scale information. EMD is a signal-decomposition method that generates a number of intrinsic mode functions (IMFs) that display characteristic timescales present in the analyzed data. A distinct advantage of the EMD with respect to other signal-decomposition methods is that the input data does not need to follow any requirements like stationarity or linearity. When using EMD, fine tuning the derivation of the IMFs is possible, but not necessary for a general application. We chose not to fine tune any parameters, other than the allowed IMF periodicities. By applying this decomposition technique we aim to comprehensively explore and compare timescales present in the corona and the photosphere to the solar-wind switchback observations from PSP.

The implementation of EMD shown here is tested and discussed in de Pablos et al. (2021), where it is applied to compare coronal timescales to solar-wind observations captured by near-Earth spacecraft. We started with selection of a short and long dataset, depending on their time duration. We then extracted windows from the long dataset with the same temporal duration as the short dataset, selecting 9-hour windows from the remote-sensing data as the short dataset-and using the in situ observations as the long dataset. We utilize the implied periodicity of each IMF ($P = 2n / T$, where $n$ is the number of maxima and minima of the IMF and $T$ is its temporal duration) to filter out timescales that were not of interest.

**Figure 11:**
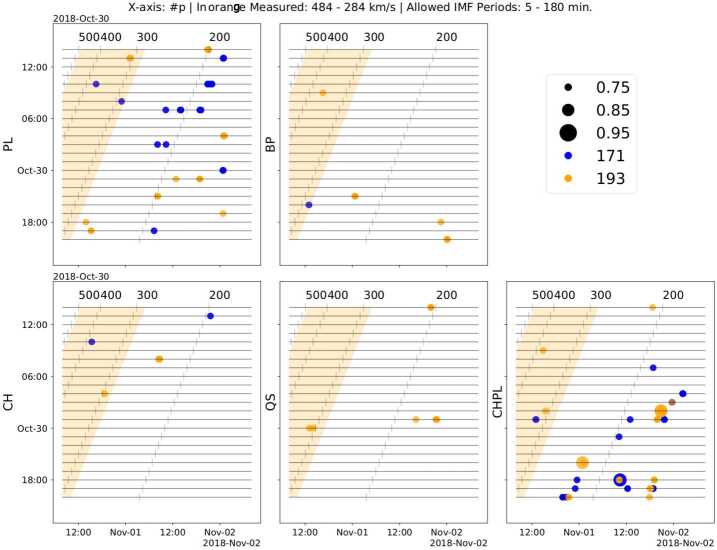
Results for correlation of IMFs generated from 9-hour windows SDO/AIA lightcurves against the proton-number density for all investigated regions. We show time of observation at PSP on the $x$-axis and time of observation in SDO/AIA on the $y$-axis. The *orange shaded band* indicates possible emission and arrival times if solar wind from the corona moves toward PSP with a constant radial proton velocity within the measured bounds at PSP of 284 to 484 km s^−1^. The size of the *circles* is directly proportional to the strength of the correlation of each 9-hour window of PSP data to the SDO/AIA data. The *circle color is blue* when the correlation is found with the 171-Å passband, and *orange* when the correlation is found with the 193-Å passband. The *orange shaded region* displayed in all panels is provided as a reference, based on minimum and maximum solar-wind velocities found in the period, indicating the expected arrival time of the signatures if they were propagating at the measured velocity throughout. The *numbers o*n top of each of the panels show other constant propagation speeds from the corona to PSP in km s^−1^.

In Figure 11, we show the times and locations of correlations between 9-hour windows of the SDO/AIA data and 9-hour windows of the PSP proton-density observations. For each of the panels, we considered IMFs with periodicities between 5 and 180 minutes, and show a circle of size relative to the strength of the correlation, colored by the relevant wavelength. We would expect the strongest correlations to be embedded within the orange band if the solar wind was propagating at a constant velocity from its coronal height to PSP, and indicate slower propagation speeds in the numbers on top of each panel. We show the correlation results of other in situ parameters with the same coronal lightcurves in the Appendix. These all show the stronger, most numerous correlations against the plume and coronal-hole plume regions.

We find the strongest correlations for the lightcurves extracted from the plume and CH plume regions, for different propagation speeds and different times for both PSP and SDO/AIA. Most are found against the 171-Å passband, with only several strong correlations with the 193-Å observations. The lightcurve extracted from the plume region shows coronal timescales being encountered in PSP number-density observations with an average solar-wind speed of 200 km s^−1^, or 50% slower than the average measured radial velocity, with coronal features released between 07:00 UT and 14:00 UT on 30 October 2018 being encountered in PSP between 1 November 2018 12:00 UT and 2 November 2018 00:00 UT. The weaker, less prevalent correlations found for lightcurves extracted from the coronal bright point, coronal hole, and quiet-Sun regions (CBP, CH, QS) do not follow any pattern, instead they are spread across different implied propagation velocities and different emission times.

#### SDO/HMI – PSP Temporal Correlation

Additionally to correlating with the coronal measurements, we compared the PSP solar-wind observations with SDO/HMI photospheric observations, with the intention to determine whether changes in total unsigned and coronal bipole flux at the footpoints of the stream would be reflected in PSP magnetic-field measurements as large-scale oscillations. We applied the same algorithm as in Section 3.1.2, correlating periodicities (IMFs) observed in windows of a long dataset to the periodicities found in a short dataset. We took the long dataset to be the in situ solar-wind observations, and the short dataset to be 9-hour windows of coronal magnetic-field measurements, as processed into total unsigned and bipole flux. We averaged down the PSP observations to the 12-minute SDO/HMI cadence and compared 9-hour portions of it to each of the 9-hour photospheric-measurement windows.

**Figure 12:**
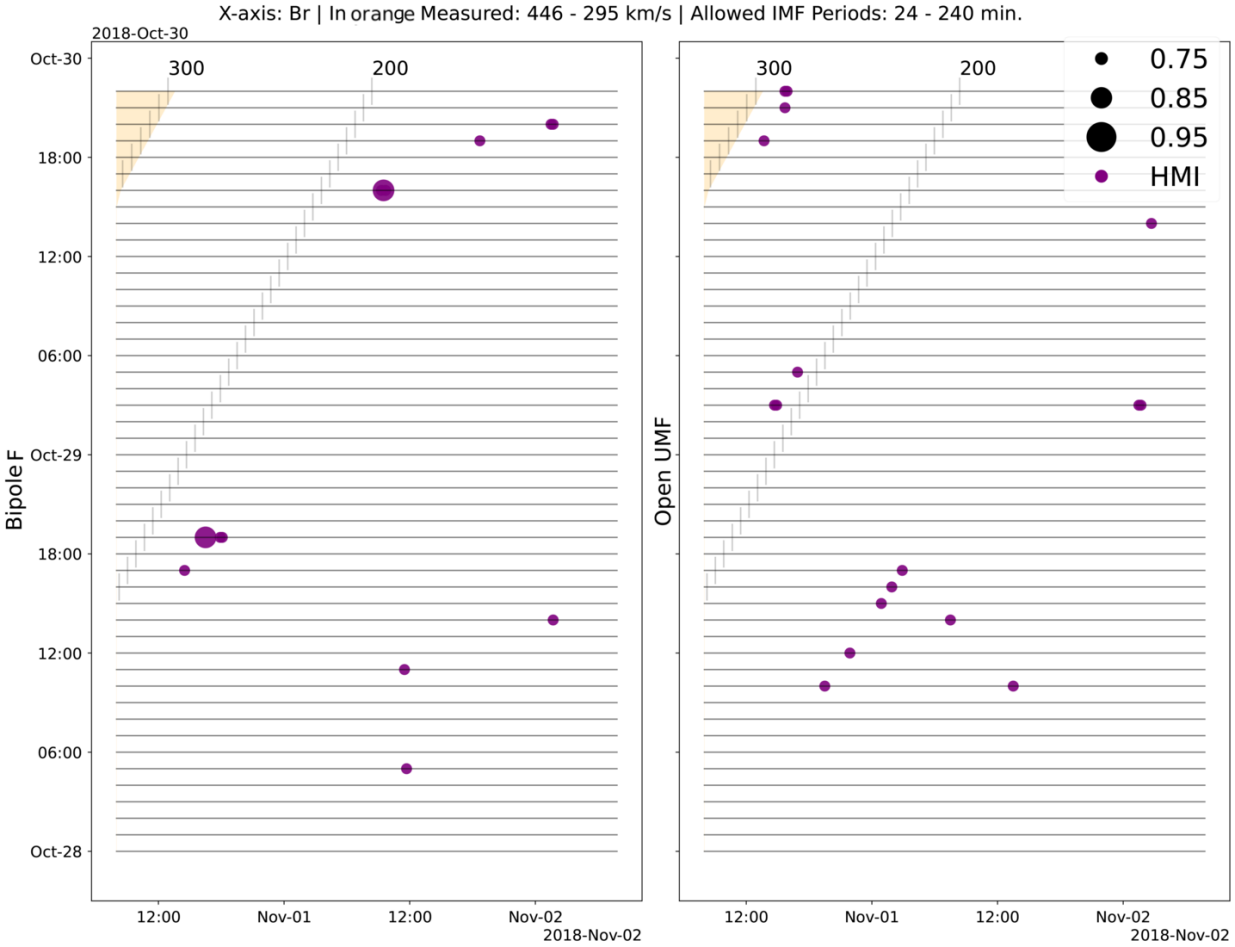
Correlation results for SDO/HMI measurements against the solar-wind radial magnetic-field component for 9-hour windows of measurements. In the *left panel* we show the correlation against the bipole flux, and in the *right panel* we show the correlation against the total unsigned magnetic flux, with the size of the *circle* being directly proportional to the strength of the correlation. The *numbers on top and vertical lines* indicate the necessary constant ballistic propagation speed for plasma to reach the spacecraft in km s^−1^, and the *orange bands* indicate the measured velocities at PSP. IMFs used for correlation must display an implied periodicity between 24 and 240 minutes.

In Figure 12 we show the EMD correlation results for 9-hour windows of the bipole flux and total unsigned magnetic flux (right) from SDO/HMI observations against scaled radial magnetic-field observations from PSP. We highlight in orange the required constant propagation velocity for the plasma to reach PSP, given an emission time equal to the midpoint of each of the SDO/HMI 9-hour windows, with the black vertical lines and numbers on top indicating other propagation velocities in km s^−1^. The purple dots indicate times with high correlation for IMFs with periodicities between 24 and 240 minutes.

For the bipole flux, we see that a maximum IMF correlation of 0.85 is reached for average propagation speeds close to 190 km s^−1^, with both very strong correlations being found for the same average propagation speed, in spite of a time difference of 20 hours in terms of the release ($y$-axis). No strong correlations are found for average propagation velocities above 200 km s^−1^. For the total unsigned flux, we note three diagonal clusters of strong correlations that agree on the average propagation velocity, with one closer to 150 km s^−1^, another to 210 km s^−1^, and the fastest one close to 290 km s^−1^. Both panels show sporadic strong correlations with no evident pattern, for very slow implied propagation speeds. These values are 40 to 55 $\%$ the average solar-wind speed measured by SWEAP at PSP.

### Spatial Analysis

In addition to the temporal analysis we performed spatial analysis of features captured in SDO/AIA, to correlate them with the PSP solar-wind observations.

We investigated the large-scale dynamics of the small CH total unsigned flux and bright-point flux within the coronal hole over several days as captured by SDO/HMI and correlated spatial signatures through a spatial Fourier transformation. To achieve this, the full images were spatially apodized using a two-dimensional cosine-bell taper of width 45 pixels applied to each edge, Fourier transformed in time and space. We then applied eight spatial Fourier filters, each of which had a Gaussian profile. The mean and standard deviation of the profiles were $(\mu ,\sigma )= (736.08, 515.26)$, (220.82, 234.45), (103.59, 117.22), (53.09, 50.50), (26.89, 26.19), (13.53, 13.35), (6.79, 6.74), and (3.40, 3.39) Mm.

Once filtered, the resulting eight data cubes in the $k-\omega $ space were inverse-Fourier transformed. Thus, we ended up with eight image time series where each time series represented the evolution of a specific spatial scale of events in the corona. We extracted the temporal evolution of the intensity of the brightest pixel found in each filtered image time series and generated a 1D array. This 1D array represented the amplitude of the strongest (or brightest) events for those of a given spatial scale and its temporal variation. We cautiously note that the maximum amplitudes from different times may not coincide at the same spatial location in this analysis.

**Figure 13:**
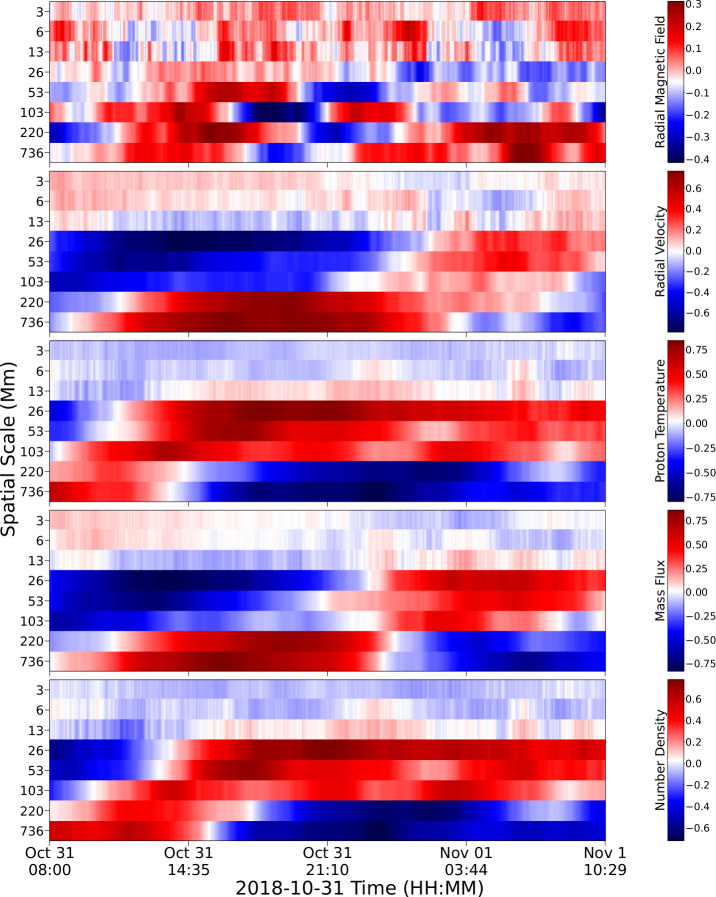
Temporal evolution of Pearson correlation coefficient for five measured in situ parameters: (top to bottom) radial magnetic field, radial velocity, proton temperature, mass flux, and proton-number density, against the SDO/AIA 193-Å image timeseries classified according to the spatial scale length.

Next, we compared this 1D array of maximum intensities with respect to time against the evolution of the PSP variables. As the PSP variables were described by a time series of 2880 times steps (with each time step being separated by 1 minute) and the SDO/AIA 193-Å images consisted of a time series of 1300 time steps beginning from 29 October 2018 at 21:50:09.35 UT and ending at 30 October 2018 at 19:30:09.35 UT, we ran a window of the size of the SDO/AIA time series on the PSP time series in order to find the Pearson correlation coefficient between these two variables. We show the resulting set of 2D images where the correlation coefficient varies with time and spatial filter in Figure 13.

We determined the spatial scale where the maximum correlation was found and its temporal location. This is illustrated in Table 1 that shows that both the radial velocity and mass flux exhibit a strong correlation of + 0.76 and + 0.86, respectively, with events corresponding to the largest spatial scale with $\mu = 736.08$ Mm. To our understanding, such a large spatial scale indicates global hemispherical events, which could be associated with the latitudinal extension of the pseudostreamer found in our PFSS model shown in Figure 4; this was a very stable coronal structure that persisted along CRs 2209 and 2210, i.e., during PSP’s time of connectivity to the small CH. This structure had been modeled and studied by Pinto et al. (2021), where in Figure 7 they show the path of the PSP footpoints, and then discuss pseudostreamer connectivity to PSP, as there are indications of the spacecraft receiving material from several pseudostreamers across PSP encounters. On the other hand, the proton temperature and the proton-number density are strongly correlated, having a correlation coefficient of $+0.84$ and $+0.78$, respectively, with events of relatively much smaller spatial scale correspondent to $\mu = 26.89$ Mm. This spatial scale is more representative of bright coronal loops seen in the intensity image of the SDO/AIA 193-Å channel. However, the radial magnetic field does not show strong correlation as other variables do. The maximum correlation coefficient for the radial magnetic field is found to be only −0.42 for a relatively intermediate spatial scale of $\mu = 103.59$ Mm, which seems to be correlated with the global hemispherical events similar to the radial velocity and mass flux.

**Table 1:** List of maximum Pearson correlation coefficient for radial magnetic field, radial proton velocity, mass flux, proton temperature, and proton-number density vs SDO/AIA 193-Å image time series with the corresponding spatial scale and temporal location.

PSP Variable	$r_{\mathrm{max}}$	sign($r_{\mathrm{max}}$)	Spatial Scale (Mm)	$T_{\mathrm{max}}$
$B_{\mathrm{r}}$	0.42	−	103.59	Oct 31 18:11 UT
$V_{\mathrm{r}}$	0.76	+	736.08	Oct 31 16:17 UT
$M_{\mathrm{f}}$	0.86	+	736.08	Oct 31 16:15 UT
$T_{\mathrm{p}}$	0.84	+	26.89	Oct 31 17:53 UT
$n_{\mathrm{p}}$	0.78	+	26.89	Oct 31 20:41 UT

Table 1 also shows the timestamps for each in situ variable when the correlation is maximum. The timestamps of maximum correlation for the radial velocity and mass flux are nearly coincident, on 31 October 2018 at 16:15 UT, while the timestamps of maximum correlation for the proton temperature and the proton-number density fall within the range of 31 October at 17:53 UT to 31 October 2018 at 20:41 UT. The radial magnetic field also reaches its maximum correlation within this range. This suggests that the events on the small CH observed by SDO/AIA (within the time range from 29 October 2019 at 21:50 UT to 30 October 2018 at 19:30 UT) are strongly correlated with all five PSP variables measured during the last 8 hours of 31 October 2018. As is evident from Figure 8, this is the time when the small CH total unsigned magnetic flux reaches its maximum and the total unsigned magnetic flux reaches its minimum. In addition, the time interval of the maximum correlation between PSP and SDO/AIA 193-Å passband suggest an information propagation speed of ≈ 275 km s^−1^, which falls within the slow solar-wind speed and close to the minimum wind velocities (≈ 284 km s^−1^) found in the PSP radial-velocity measurements.

## Discussion and Conclusions

In this article, we investigate observations that contextualize and explain the solar wind observed during the Parker Solar Probe first perihelion. We present the magnetic-field configuration one Carrington rotation prior and during the encounter, which displays a long-lived pseudostreamer and a strongly evolving magnetic flux and coronal-hole boundaries within the region. We also show a large number of upflows identified with the EIS spectrometer, which are likely to play a part in the ejected solar wind and switchback formation.

Previous work (Macneil et al., 2020) has demonstrated that the occurrence rate of switchbacks generated within the solar wind is directly proportional to distance from the Sun beyond 0.3 AU. If this trend can be extrapolated inside 0.3 AU, we expect events in the coronal footpoints of the PSP spacecraft to play an important role in switchback formation and frequency, as the spacecraft captures solar wind at 0.2 AU.

We extract coronal observations around the time of the PSP perihelion from several distinct regions around the spacecraft footpoint and correlate these measurements directly to find very weak correlation (≤ 0.6) for all investigated parameters and regions. By then employing empirical mode decomposition, we remove frequencies that were likely noise, or background oscillations, and compare the remainder signal using their intrinsic mode functions. We describe the correlations against the same set of in situ parameters and find that compared to the expected arrival time given pure ballistic backmapping, our results imply that the solar wind arrives later than expected, with an average solar-wind speed lower than the ballistic propagation, of about 200 km s^−1^ instead of 400 km s^−1^. This finding agrees with the early-stage acceleration theory and observations of the solar wind, as coronal outflows of ∼ 100 km s^−1^ are primarily accelerated between 1.5 and 6 R_⊙_ (see, e.g., Figure 7 from Bemporad, 2017). The two parameters most strongly correlated when employing EMD are the proton-number density and the coronal-hole plume and plume regions.

When comparing the evolution of open and coronal bright-point photospheric fluxes to the radial magnetic field and mass flux, we also find that the temporal signatures match solar wind arriving later than expected, implying slower average solar-wind propagation velocities of about 180 km s^−1^.

We perform a spatial Fourier transformation of the coronal observations and correlate the results against the outflowing solar-wind measurements. We find that large-scale events in the corona ($\mu =736.08$ Mm) are strongly correlated with the radial proton velocity and the mass flux, indicating global hemispherical structures (see the global coronal model in Figure 4) have an influence on the solar-wind speed and the outflowing mass. The proton temperature and number density are found to instead be well correlated to small spatial scale events of $\mu =26.89$ Mm. This scale is similar to that of bright coronal loops in AIA 193 Å and the upflows found within the EIS observations, as well as to the spatial scale seen in switchbacks (Bale et al., 2021). The necessary average solar-wind proton velocity for these timings to match would be 275 km s^−1^, closer to the minimum speeds measured by the spacecraft but still requiring acceleration.

We have found evidence of correlation under the assumption of slow accelerating solar wind, as well as evidence for spatial structures on the Sun correlating well to in situ data. Since the spatial correlation technique shows a necessary proton velocity closer to that measured at PSP than either of the time-based correlations, we believe that future studies on coronal influence on solar-wind release would be more complete by considering the methodology shown here, investigating other PSP encounters for which the source regions are well identified.

## Figures and Tables

**Figure 14:**
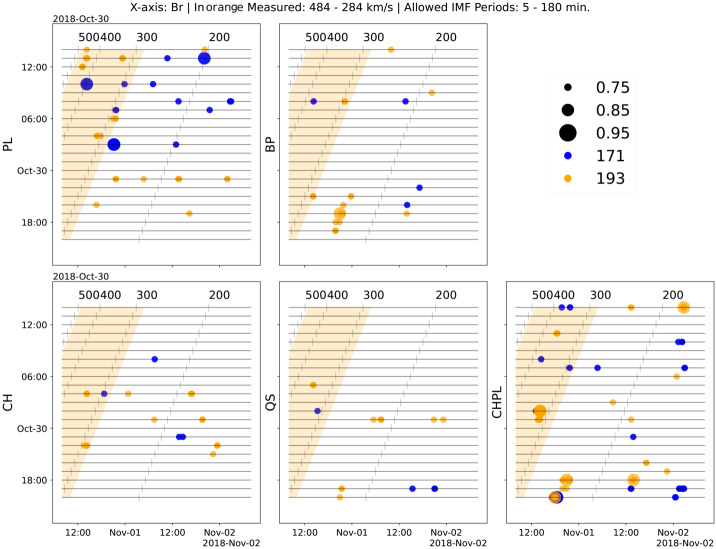
Results for correlation of IMFs generated from 9-hour windows SDO/AIA lightcurves against the radial magnetic field, for all investigated regions. We show time of observation at PSP on the $x$-axis and time of observation in SDO/AIA on the $y$-axis. The size of the circles is directly proportional to the strength of the correlation of each 9-hour window of PSP data to the SDO/AIA data. The circle color is blue when the correlation is found with the 171-Å passband, and orange when the correlation is found with the 193-Å passband.

**Figure 15:**
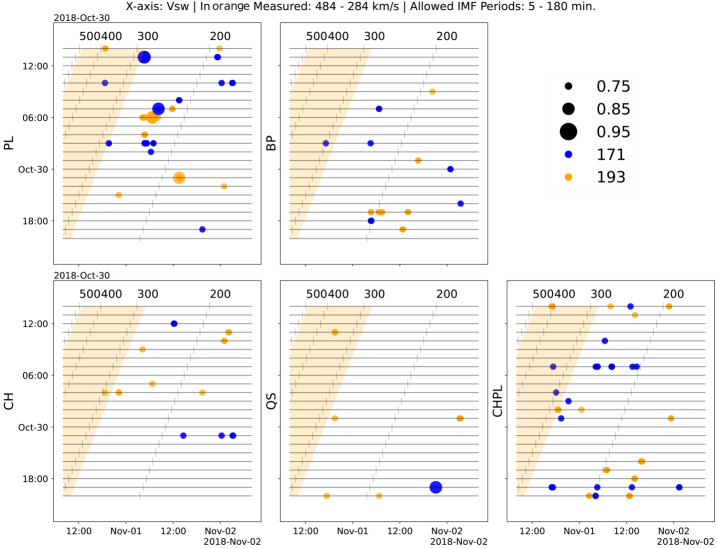
Same format as Figure 14 for the radial proton velocity as measured in PSP.

**Figure 16:**
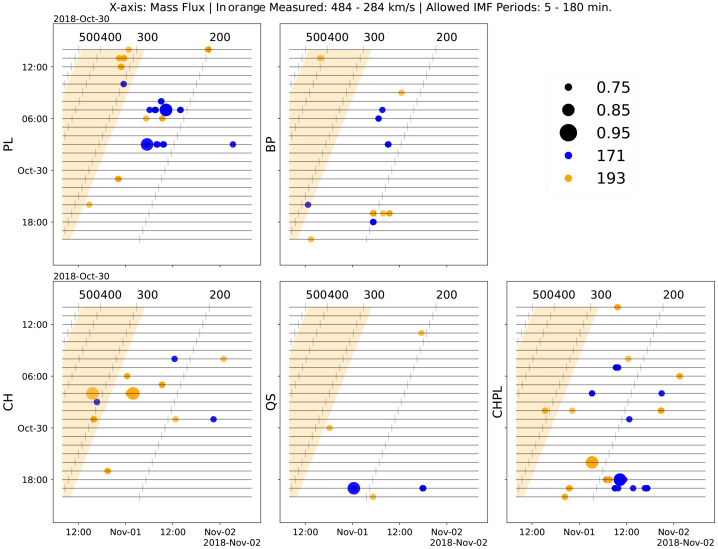
Same format as Figure 14 for the mass flux as measured in PSP.

**Figure 17:**
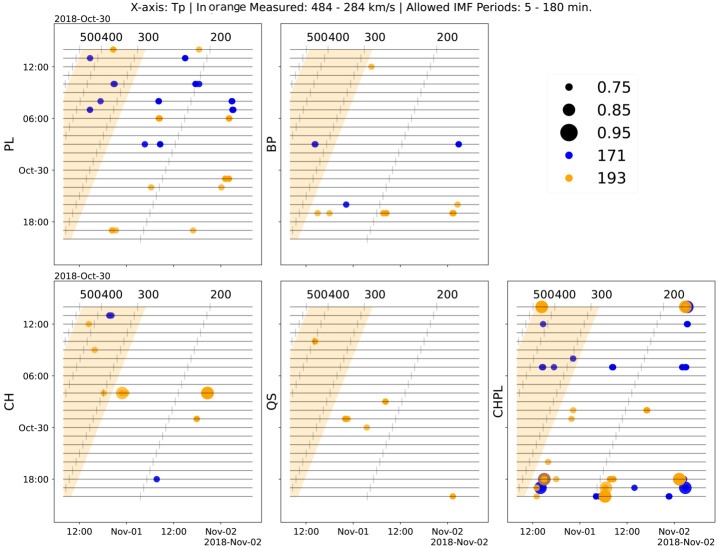
Same format as Figure 14 for the proton temperature as measured in PSP.

## Data Availability

Data used in this study is publicly available at cdaweb.gsfc.nasa.gov/index.html/ (PSP) and jsoc.stanford.edu (SDO).

## Appendix: EMD Correlation Results

In this Appendix, we include the EMD correlation results for in situ variables other than the proton density that we show in Figure 14. The remote-sensing regions that are used to extract the data for correlation are described in Figure 9a.

In Figure 14 we show the EMD correlation results for the radial magnetic field and the SDO/AIA lightcurves. The largest number of correlations is found for the plume and coronal-hole plume regions, with the other regions not finding as cohesive results in terms of correlations. The strongest correlation is for the coronal-hole plume measurements in the 193-Å passband, which matches with a propagation velocity of 290 km s^−1^, similar to the minimum observed proton radial velocity from PSP. Notably, both in the plume and coronal-hole plume there are high correlations within the orange band and therefore within the margins of a constant velocity ballistic backmapping.

We show the EMD correlation results for the solar-wind radial velocity in Figure 15. The regions that display the larger number of correlations are the plume and coronal-hole plume, and we find less consistency across wavelengths than in the radial magnetic-field case that we show in Figure 14.

In Figure 16 we show the EMD correlation results for the remote-sensing observations against the solar-wind mass flux. We find that the plume and coronal-hole regions agree with respect to the timing of high correlations. The largest number of correlations is found for the plume and coronal-hole plume regions, against the 171-Å passband. The highest correlation is found for the coronal hole, for an implied velocity of ≈ 260 km s^−1^.

We show the EMD correlation results of the proton temperature against the remote-sensing regions in Figure 17. The regions with the largest number of strong correlations are the plume and coronal-hole plume. While more strong correlations are found for the later half of the 171-Å passband, they are instead found in the earlier half of the 193-Å observations. No correlations above 0.85 are found for these parameters.
